# Development and Assessment of Traditional and Innovative Media to Reduce Individual HIV/AIDS-Related Stigma Attitudes and Beliefs in India

**DOI:** 10.3389/fpubh.2013.00021

**Published:** 2013-07-22

**Authors:** Caricia Catalani, Diego Castaneda, Freya Spielberg

**Affiliations:** ^1^School of Public Health, University of California at Berkeley, San Francisco, CA, USA; ^2^Innovative Support to Emergencies, Diseases, and Disasters (InSTEDD), Sunnyvale, CA, USA; ^3^Department of Psychiatry, University of California, San Francisco, CA, USA; ^4^School of Public Health, George Washington University, Washington, DC, USA

**Keywords:** stigma, HIV, AIDS, media, video, information technology, computer, India

## Abstract

Although stigma is considered a major barrier to effective response to the HIV/AIDS epidemic, there is a lack of evidence on effective interventions. This media intervention took place among key HIV-vulnerable communities in Southern India. Two HIV stigma videos were created using techniques from traditional film production and new media digital storytelling. A series of 16 focus group discussions were held in 4 rural and 4 urban sites in South India, with specific groups for sex workers, men who have sex with men, young married women, and others. Focus groups with viewers of the traditional film (8 focus groups, 80 participants) and viewers of the new media production (8 focus groups, 69 participants) revealed the mechanisms through which storyline, characters, and esthetics influence viewers’ attitudes and beliefs about stigma. A comparative pre-/post-survey showed that audiences of both videos significantly improved their stigma scores. We found that a simple illustrated video, produced on a limited budget by amateurs, and a feature film, produced with an ample budget by professionals, elicited similar responses from audiences and similar positive short-term outcomes on stigma.

## Introduction

Since the beginning of the HIV epidemic, stigma has been identified as a major barrier to HIV prevention (1–4). Among the many challenges posed by stigma, it has been directly attributed to lower uptake of HIV prevention services, testing, and counseling (1, 5–7). Despite its importance, there is little strong evidence on interventions that address stigma (8). Most efforts have focused primarily on information dissemination, empathy induction, counseling, and cognitive behavioral therapy. These interventions have been evaluated with little attention to rigor if evaluated at all (1). Moreover, the stigma intervention literature offers a limited practical understanding of how programmatic leaders might work to change stigma beliefs as a part of increasing demand for prevention services within key communities (9).

Among the limited evidence available, there is some indication that media can be used as a vehicle to effectively convey HIV education and achieve reductions in HIV-related stigma. O’Leary et al. (10), for example, found that exposure to an HIV stigma related storyline appearing in the popular soap opera “The Bold and the Beautiful” was associated with significantly lower levels of HIV stigma. Other practitioners have tried to apply these promising findings to their own media interventions; however, most have found limited success (11).

Today, with growing access to simple and affordable media production tools around the world, HIV advocates and educators in both low- and high-resource settings are looking to take advantage of new technological opportunities and produce their own effective media campaigns. To provide evidence to support and guide this opportunity, we conducted a mixed methods study to examine the following questions: (1) How can media be developed to address HIV-related stigma? (2) How might media storyline, characters, and esthetics influence audience attitudes and beliefs? and (3) How effective are these media in changing attitudes and beliefs of viewers from key populations?

## Materials and Methods

This project took part in three phases: the development of a feature film *Prarambha* (The Beginning), the development of an illustrated stigma video, and the subsequent pilot testing of both media.

### Development of feature film

The feature film, *Prarambha* (The Beginning), was produced by Mira Nair with the aim of generating awareness about HIV/AIDS and related stigma. The film was directed by Santosh Sivan in a joint initiative of Mirabai Films with support from Avahan, Richard Gere’s AIDS Foundation, and the Bill and Melinda Gates Foundation. Shot entirely on location in Mysore, the film depicts everyday sights and sounds of South India and features popular local actor Prabhu Deva in the principal part of a truck driver, Ramu. Ramu befriends a child, Kittu, whose estranged parents were diagnosed HIV+ and who is himself HIV+, resulting in his expulsion from primary school. As their adventures together unfold, Ramu champions the issue of overcoming HIV-related stigma and discrimination in public schools. Despite his HIV-status, Kittu is readmitted to school among a community of students, parents, teachers, and administrators who are more accepting and informed about HIV. The basic storyline of *Prarambha*, and the illustrated video it inspired, are depicted in Table 1.

**Table 1:** **Storyline and screenshots**.

Stigma story	Feature film	Illustrated Video
A young boy, Kittu, approaches a truck driver, Ramu, and asks to take him to Mysore to find his mother.	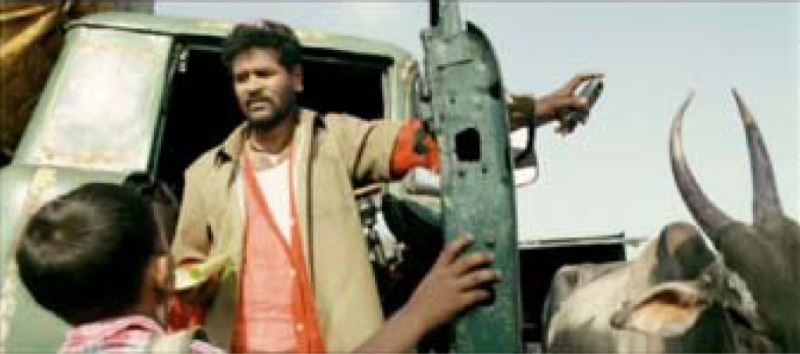	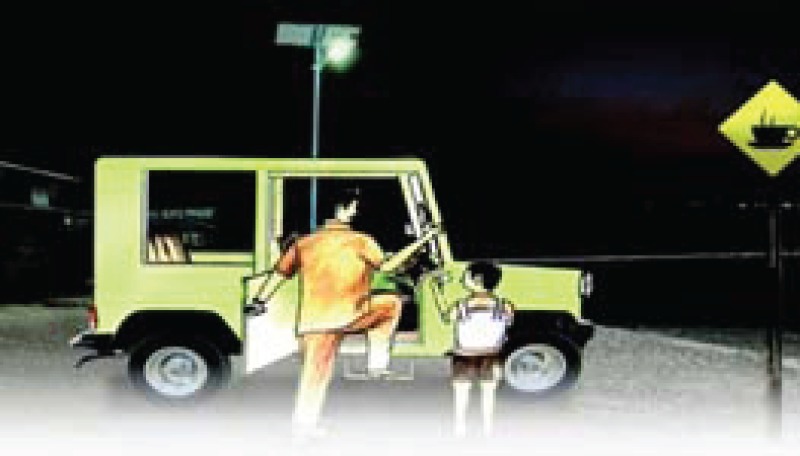
After searching, they find Kittu’s mother in a hospital, where she is in the end stages of AIDS.	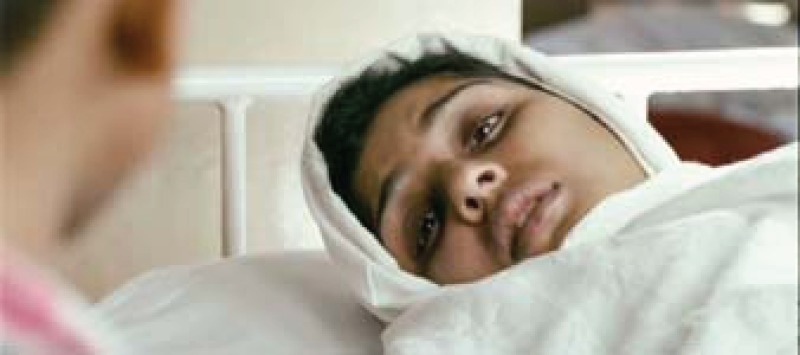	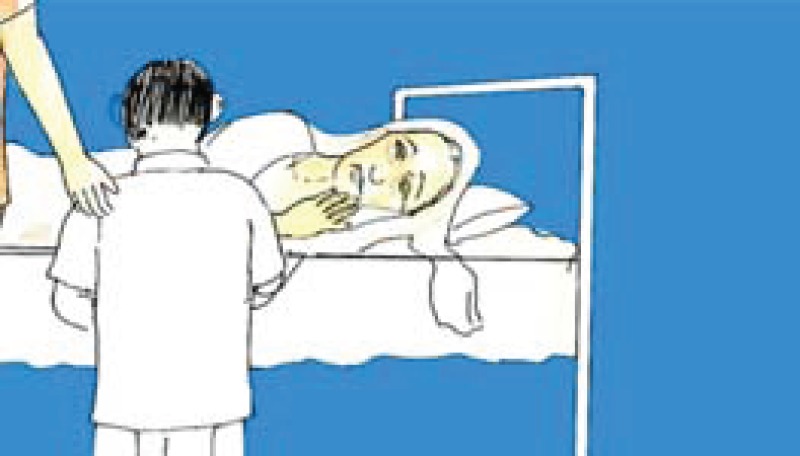
Kittu tells Ramu that he was ostracized and forced to leave school due to his and his parents HIV-positive diagnosis.	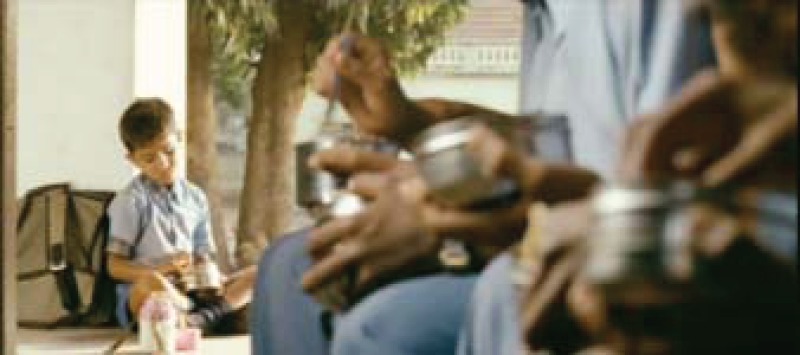	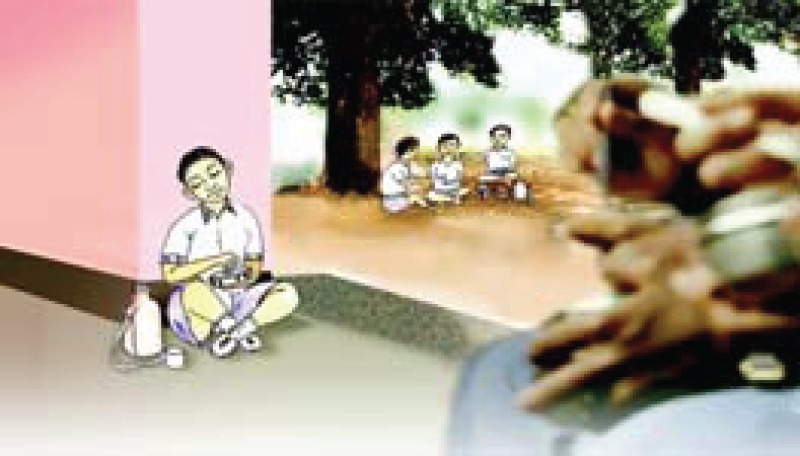
Ramu petitions the principal to fight for Kittu’s return to school.	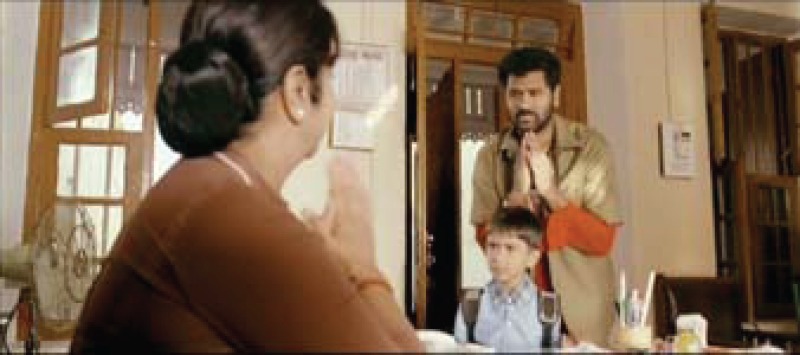	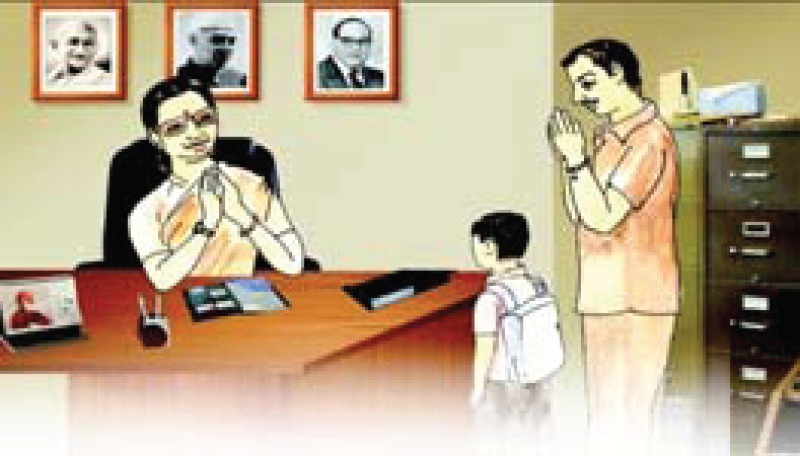
Principal becomes a key spokeswoman for HIV awareness, HIV education, and the fight against HIV-related stigma.	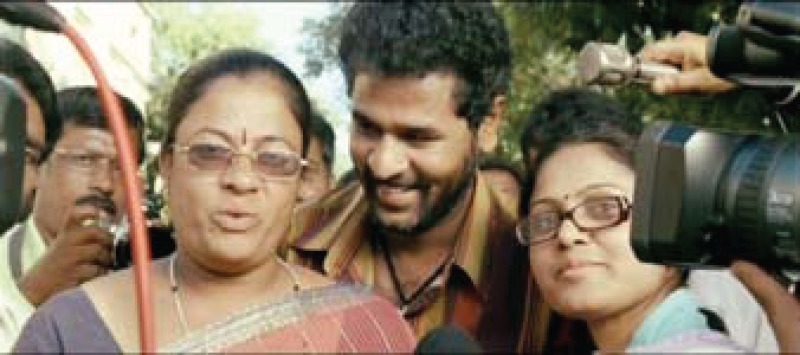	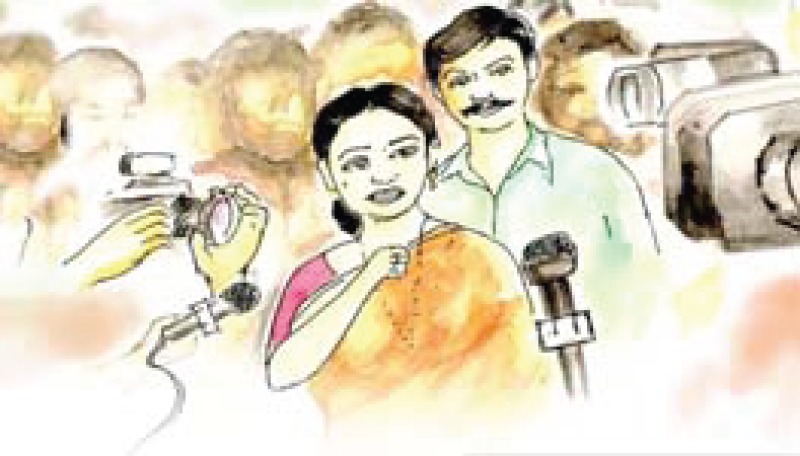
Kittu returns to school, with the blessing of administration and other parents, where other children embrace him.	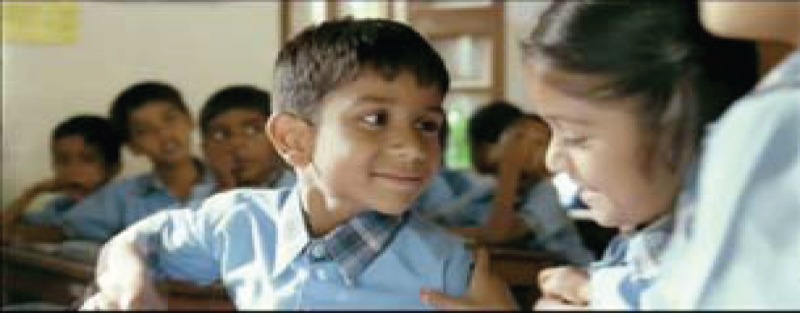	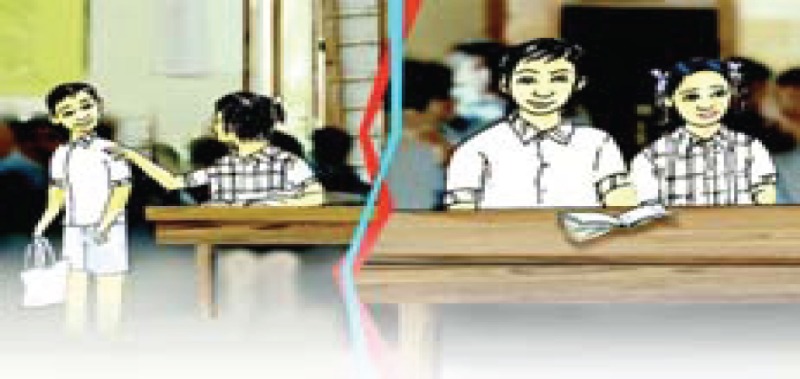

*Prarambha* was developed and produced during a span of approximately 1 year from 2006 to 2007 in three stages: Pre-production, production, and distribution. Producers focused on making a narrative film, using the same standards and techniques as other high-end projects. They explained, “We were focused on making a film, just like any other film, and not a PSA.” As such, staff at Mirabai emphasized the creation of an engaging and true to life story (12).

Pre-production included a myriad of activities from fundraising and contract negotiation, to recruitment of contributors and coordination of their schedules, to storyline development and screenwriting. The film was supported by the Gates Foundation for under $250,000, a budget made possible by severely discounted rates from some of India’s most revered filmmakers and actors. Staff noted that recruiting directors and performers was not seemingly hampered by HIV-related stigma, but rather facilitated by the rising threat of HIV in India (12). The most challenging aspect of pre-production was the coordination of filmmaker and actor schedules, given the short duration of the shoot (1 week), and demanding agendas of Bollywood professionals.

During the production stage, Prarambha was filmed on 35 mm over 1 week. Although there were two locations that declined to appear in the film, which staff perceived to be related to stigma, Mirabai staff mostly found an outpouring of local support for a film about HIV in Southern India. The short shooting period facilitated participation from staff and sites, making it easier to negotiate lower contract costs. Editing, finalizing, and wrapping the film required 10 months, as Mirabai staff rushed to complete the project before the beginning of the international film festival season.

The final stage, distribution, began with the international premiere of Prarambha at the 2007 Toronto International Film Festival. Mirabai worked to distribute the film in India and internationally through film festivals, television broadcasting, online screening, and community screenings through partnerships with high schools, bus companies, airlines, and clinics and hospitals. Distribution is ongoing and, as of November 2010, Mirabai distributors estimated that three million viewers had seen Prarambha.

### Development of illustrated video

To be able to compare the processes and impacts of a similar story produced with a limited budget and novice media production team, our team created a simple illustrated video based on Prarambha. This illustrated video was developed in Bangalore during four production stages by a team of health communication experts from South India and the United States, an artist from India, four community translators representing four South Indian languages, and eight voiceover artists to depict female and male voices in four local languages. During the first stage of production, the most essential aspects of Prarambha’s storyline was reformulated into a simple comic style digital story with hand-drawn images and script for voiceover. Second, the team constructed a simple sound recording booth in an office closet, recruited voiceover artists, invited script feedback from voiceover artists, and recorded the script with an H2 Zoom audio recorder. Finally, after a 1-day video editing workshop, the team combined the artists’ images with corresponding audio recordings using Final Cut Express video editing software (2010, Apple, Inc., CA, USA). Using the medium of an illustrated digital story, the team was able to create videos in four different local languages by inserting different language audio tracks during the editing process. Production, which began with an established storyline, took approximately 2 months and cost $8,000, including staff time.

### Delivery and viewing of media

Recruitment and participation in this media study took place during an 8-week period in summer and fall of 2010. Participants were recruited with the assistance of community-based organizations and community clinics in four different rural and four different urban field locations using purposeful sampling of the following specific population subgroups: female sex workers, men who have sex with men (MSM), young married women, and married men. Men and women who agreed to participate in our media study were randomly selected into either the feature film or illustrated video group. Due to social sensitivity around issues of sexual health, sessions were held separately among women and men.

Participants arrived at a partnering clinic or community-based organization at a predetermined date and time in groups of 6–12 people. Upon participant arrival, research staff administered a demographic and pre-survey in one of three preferred local languages in a private setting with each participant. Together, the group of participants viewed either the feature film or illustrated video. Facilitators used a laptop, projector, and speakers to screen media. As is described in greater detail below, facilitators then led a focus group discussion. At the close of each session, research staff administered a post-survey with each participant, as before. Each session lasted approximately 1.5 h, with either 11 min for viewing the feature film or 3 min for viewing the illustrated video, and approximately 45 min dedicated to post-viewing group discussion. This one-time intervention did not include any follow-up sessions. Given the nature of this study, it was not possible to blind participants to study condition assignment. Based on recommendations from local partners, participants were not remunerated, however they were served a lunch and provided with a gift valuing $2–4 USD.

### Evaluation of feature film and illustrated video

#### Data collection

The human subjects protection committee of RTI International approved this study and all participants provided informed consent. As described, audiences engaged in focus group discussions following the screening of either the feature film or illustrated video. Within the feature film set, a total of 80 people participated in 8 focus groups. Within the illustrated video set, a total of 69 people participated in 8 focus groups. A detailed description of focus group participant characteristics is provided in Table 2.

**Table 2:** **Participant demographics**.

Overall, *N* = 149	Feature film, *n* = 80	Illustrated video, *n* = 69
**GENDER^1^**		
Women	65	59
Men	35	41
**AGE^1^ (YEARS)**		
16–25	53	51
26–40	35	40
40+	12	9
**COMPLETED EDUCATION^1^ (YEARS)**		
1–5	15	23
6–12	70	64
12+	15	13
**NEVER USED A COMPUTER BEFORE^1^**	81	78
**EARNED MONEY FOR WORKING IN PAST YEAR^1^**	60	61

*^1^The chi-square statistic was not significant for any of the participant characteristics at a significance level of 0.05*.

Two local female physicians conducted each of the focus groups in Kannada, the primary language of Karnataka state. After screening either the feature film or illustrated video, these experienced facilitators led discussions based on a semi-structured guide developed in close coordination in order to minimize facilitator bias. Discussion topics focused on media characteristics that have been shown by others to influence viewers’ attitudes and beliefs (13–15), including: (1) Cultural relevance and believability of storyline, (2) clarity, comprehension, and knowledge of key stigma educational messages, (3) perceived susceptibility, relevance, and attitudes related to stigma, (4) identification and empathy with main characters, and (5) video esthetics.

The pre- and post-test surveys included a number of statements that were taken from previously validated questions designed to capture proven stigma constructs, “negative judgments about people living with HIV,” and “fear of transmission from casual contact” (16–18). Participants were asked whether they “agreed,” “were not sure,” or “disagreed” with four statements measuring “*negative judgments about people living HIV/AIDS*”(two in the pre-test and two in the post-test) and six statements measuring “*fear of transmission through casual contact*”(two in pre-test and four in the post-test). See Table 3 for specific statements for each these constructs.

**Table 3:** **Pre-test and Post-test Wilcoxon Signed Rank Test for stigma outcome variables by treatment group**.

	Feature film				Illustrated video			
	Pre-test mean score	Post-test mean score	*z*	*p*	Pre-test mean score	Post-test mean score	*z*	*p*
Negative judgments about people living with HIV/AIDS, 1 = agree, 2 = not sure, 3 = disagree	1.96^1^	2.67^2^	−5.60	<0.001	1.98^1^	2.79^2^	−5.64	<0.001
Fear of contracting HIV from casual contact,1 = agree, 2 = not sure, 3 = disagree	2.41^3^	2.60^4^	−2.79	<0.05	2.70^3^	2.66^4^	–	ns
Overall stigma score, mean (fear of casual contact and negative judgments score)	2.20	2.70	−4.80	<0.001	2.35	2.72	−4.50	<0.001

^1^Average of scores from the following two statements: “HIV/AIDS is a punishment for bad behavior”, and “If someone has contracted HIV by having unsafe sex, it is their own fault.”

^2^Average of scores from the following two statements: “People with AIDS should be ashamed of themselves,” and “People with AIDS should be ashamed of bringing the disease into their communities.”

*^3^Average of the scores from the following two statements: “Talking to someone with HIV/AIDS puts you in danger of getting HIV/AIDS”, and “Teachers who have HIV/AIDS should be allowed to continue teaching in school” (score recoded for directionality)*.

*^4^Average of scores from the following two statements: “A person who has HIV/AIDS should not be allowed to work, to protect the people who don’t have HIV/AIDS”, and “I would feel comfortable traveling with someone who has HIV/AIDS in the same vehicle” (score recoded for directionality)*.

#### Data analysis

English-language transcripts of all focus group sessions were entered into Atlas.ti qualitative analysis software (2010, Atlas.ti Scientific Software Development Gmbh, Cologne, Germany). Four researchers developed an initial list of codes based on theoretical literature on HIV-related stigma and health communications. Two researchers systematically coded transcripts using a set of predetermined theoretical codes and integrated a more grounded approach by added to and refining codes according to emergent themes from the data (19). Researchers discussed interpretation and reconciled any differences in coding throughout the systematic coding process. Analysis of transcribed audio recordings of the sessions revealed that there were no significant variations in focus group facilitation. Atlas.ti was used to sort data by codes and supercodes, identify and create subcodes, understand differences and similarities in responses within codes, recognize relationships between codes, and create summaries of the principle concepts across focus group discussions.

Quantitative data was cleaned, managed and analyzed using SPSS version 17.0 (2008, SPSS Inc., IL, USA). A Wilcoxon Signed Rank test was run to detect statistical differences in mean “negative judgments” and “fear of transmission from casual contact” scores between the pre and post-tests. Overall stigma score was compiled by averaging the latter two scores. Data was then stratified by type of videos watched (illustrated video vs. feature film) and tested using the same statistical method. Significance testing was completed at the 0.05 level. As a missing value analysis showed that less than 10% of the sample for any given item was missing, mean imputation was used to correct for missing data.

## Results

### Qualitative themes

The primary issues discussed by participants included: (1) Cultural relevance & believability of storyline, (2) perceived susceptibility, relevance, and attitudes related to stigma, (3) identification and empathy with main characters, and (4) video esthetics. As described below, there were key differences and similarities in the discussion of these issues by participants who viewed the feature film vs. the illustrated video.

#### Cultural relevance and believability of storyline

Across and within focus groups, participant shared the opinion that the storyline seemed believable and possible. Participants often shared personal anecdotes that related to the film, telling their own stories about individuals making a difference in the lives of others or their communities and fighting for those who are vulnerable, as depicted in the videos. Interestingly, several participants in both the illustrated video and feature film focus groups tended to agree that awareness campaigns through the media have been helpful in addressing stigma. Despite these feelings of optimism, however, it was clear that the film and video struck a critical chord among many viewers, generating a discussion about incidents of stigmatization they had either heard about or personally witnessed in their communities. In terms of this theme, there were no major or enduring differences in discussion between those who viewed the feature film and illustrated video.

#### Perceived susceptibility, relevance, and attitudes related to stigma

Across the focus groups, participants agreed that stigma was a real and important issue in their communities. Participants from both film and video groups tended to deny any personal discriminatory attitudes but, rather, explained that they avoided people living with HIV due to fears about transmission. When asked about this, comments by viewers in both groups suggested that the film and video decreased their fear of transmission. For instance, one of the feature film viewers stated, “Previously we think it was spread so we don’t go to anybody’s house for dinner, don’t go near them because we are afraid. But now I am more aware and I feel happy. I will tell my neighbors to be the same way too.”

When it came to engaging participants in critical discussion about their own susceptibility to stigmatizing attitudes, it was observed that those who viewed the feature film engaged in more active debate on the issue than those who viewed the illustrated video. Within these more active discussions, participants discussed varying points of view, imparted personal anecdotes, and provided ideas for how stigma might be better addressed in communities.

#### Identification and empathy with main characters

One key message in the narrative across both film and video groups was empathy and compassion toward those who are living with HIV. This was echoed strongly across focus groups, particularly in reference to the main character, a child who is diagnosed HIV-positive. Responding similarly, a feature film viewer stated, “*Kittu is like all children and he must have the same rights*” and a illustrated video viewer said, “*He is like our children, the same as any of our children*.” Viewers of the illustrated video tended to have an especially strong emotional response to this message as compared to the feature film, some becoming very emotional about how unfair stigma is for those who are hurt by it.

However, viewers across both did not share this same empathy with Kittu’s mother, a supporting character. While Kittu was understood to be “innocent” by most viewers, some wondered if his mother was a sex worker and looked to place blame, stating “*Infection of the boy comes from the parents situation*… *this is the parents’ fault*.” Interestingly, although participants in both groups expressed dismay and sadness about Kittu’s isolation by his mother and blamed her for the situation Kittu was in, there was some sympathy and understanding for the reasons for actions. As became the focus of some discussion, one viewer stated, “*If community will accept her and all HIV patients, then his mother wouldn’t [have to] isolate the child*.”

The third character, Ramu, was viewed sympathetically by participants, who believed that he was a man with “a good heart”, who went above and beyond to help a small child. Perhaps due to his heroic nature, some participants in the illustrated group felt that Ramu was too good to be true and that most people, in their real experience, would not go to such lengths to defend another. Characteristic of this doubt, one participants said, “*Nobody can have that much of helping nature. They don’t have that much of care about others’ children*.” Participants in the film group, however, didn’t seem to have these doubts about Ramu’s helping nature, only expressing their admiration for his actions in defending Kittu’s right to go to school.

#### Esthetics and story

Participants from each group responded positively to each medium. Those who viewed the feature film tended to comment that they enjoyed seeing real sites from around Mysore, recognized well-known film actors, and appreciated some of the complexities of each character. Those who viewed the illustrated video discussed the artists’ images and the voiceover narration and found this style esthetically agreeable.

### Quantitative measures

A Wilcoxon Signed Rank test was conducted to detect differences in pre and post-test stigma (negative judgments and fear of casual contact indices) scores for viewers of the feature film and viewers of the illustrated video. For viewers of the feature film, an index measuring negative attitudes toward those with HIV/AIDS showed a positive significant change in scores (*z* = −5.60, *p* < 0.001), between the pre-test (*M* = 1.96) and the post-test (*M* = 2.67). Viewers of the illustrated video also showed significant positive changes in this index (*z* = −5.64, *p* < 0.001), showing marked improvement between the pre-test (*M* = 1.98) and post-test (*M* = 2.79). Large effect sizes were seen in both the feature film (*r* = 0.6) and illustrated video (*r* = 0.7) groups (See Table 3). For the fear of casual contact index, however, only the feature film showed a moderate significant change (*z* = −2.21, *p* < 0.05) between pre-test (*M* = 2.41) and post-test (*M* = 2.60) with a moderate effect size (*r* = 0.3).

The Wilcoxon Signed Rank test was also used to detect differences in the pre and post-test overall stigma index (average of “fear of contracting HIV from casual contact” and “negative judgments about people living with HIV” scores) between the two treatment groups. This test showed significant improvements in stigma attitudes regardless of whether they watched the feature film or illustrated video. In the film group, stigma score improved from the pre-test (*M* = 2.20) to the post-test (*M* = 2.70) (*z* = −4.8, *p* < 0.001) and showed a robust effect size (*r* = 0.5). Likewise for the illustrated video, pre-test score (*M* = 2.35) significantly increased to (*M* = 2.72) (*z* = −4.5, *p* < 0.001) with an equally robust effect size (*r* = 0.5).

## Discussion

In this study, a team of researchers and media production specialists developed and tested two media interventions to address HIV-related stigma among key populations in Southern India. Although stigma is known to have severe impact on uptake of HIV prevention and treatment interventions, the past 20 years of HIV intervention and research has provided little guidance on how to catalyze social and individual change. Our teams implemented two media development strategies: (1) a professionally produced feature film supported by a substantial budget, starring well-known celebrities and (2) an illustrated digital story created by an amateur team of HIV advocates with a severely limited budget.

Qualitative findings reveal that audiences characterized both the stigma feature film and illustrated video as having dramatic storyline, believable and culturally relevant contexts, and sympathetic characters. These traits have been described elsewhere, particularly by Fisher’s narrative theory, as being critical to effective media because (a) dramatic stories have a broad audience appeal and (b) believable and relevant stories provide “good reasons” for changing attitudes and behavior (20). Studies testing these assertions and the influence of sympathetic characters confirm that television viewers, for example, have been found to be particularly likely to respond positively to prevention messages when they are delivered dramatically by a character the viewers care about (14, 21). Adding something new to the discourse on effective media, though, this study may be among the first to explore the way that media production-value influences these fundamental traits. And, ultimately, our findings imply that professional production standards or celebrity engagement may not be as crucial to message effectiveness.

Quantitative findings demonstrate that media, even in the form of short videos, can produce significant impact on audiences, in this case decreasing HIV-related stigma attitudes and beliefs among viewers. A similar study by O’Leary et al. (10) concluded that stigma indices were reduced after viewers in Botswana watched a series of televised soap operas in which a person living with HIV was treated in a warm and non-stigmatized manner by others in their family and community. Although O’Leary and other entertainment researchers (22) argue that these effects may be, in part, due to the fact that viewers watched several professionally produced media over time, our study supports the assertion that a more limited and amateur media intervention may also have positive benefits, at least in the short-run.

Several limitations should be considered in the translation of these findings. Foremost among them is our limited definition of stigma. Stigma is understood by Goffman (23) as “an attribute that is deeply discrediting” and that reduces a person “from a whole and usual person to a tainted, discounted one” (p. 3). Parker and Aggleton (24) point out that stigma is also a social phenomena that should be understood within the context of broader structural inequalities and systems of power and oppression. In agreement, Link and Phelan (25) propose that “stigma exists when elements of labeling, stereotyping, separating, status loss, and discrimination co-occur in a power situation that allows these processes to unfold” (p. 382). As such, stigma overlaps with other forms of discrimination, such as racism, homophobia, classism, and sexism, disproportionately affecting socially vulnerable groups (2, 26–28). Our study did not examine the influence of these inter-related forms of stigma, perhaps missing the target in terms of offering an intervention that addresses the roots of stigma.

Although these results are suggestive, it is important to note that causal inferences cannot be drawn from this non-randomized study. Participants for this study were recruited through purposeful sampling with the assistance of local organizations, meaning that the findings are not generalizable beyond this group. Stratifying our populations into separate groups (married women vs. sex workers) could have been useful to detect whether any differences in how particular populations view HIV/AIDS affect outcomes, however we lacked sufficient numbers to make this statistically feasible. Although we compared two varying media strategies, we did not include a control group for comparison with no media intervention. A difficulty in media studies is detecting whether changes potentially stemming from the media intervention stay with the participant (29). A longitudinal study where participants are followed and retested would allow for more robust interpretation. Finally, it is essential to consider the possibility that the focus group discussion itself has an effect on outcomes. Engaging participants in a group discussion about stigma after viewing may impact knowledge, attitude, and behaviors more than viewing alone. Therefore, we cannot make conclusions about the potential impact of interventions that are delivered through mass media channels or online media sites.

## Conclusion

This mixed methods study describes how media can be developed to address HIV stigma, the mechanisms through which media storyline, characters, and esthetics influence audience attitudes and beliefs, and the comparative effectiveness of two media interventions among HIV key populations in Southern India. We found that our feature film and simple illustrated video were both characterized by viewers as having a dramatic storyline, believable and culturally relevant contexts, and sympathetic characters. Perhaps due in part to these traits, audiences had lower scores of HIV-related stigma attitudes and beliefs after viewing. Both qualitative and quantitative findings confirm that there was no meaningful difference in the ways the audiences perceived the two media or the changes observed in audience attitudes and beliefs. This suggest that innovative media production techniques can be used by amateurs with minimal training and funding to produce effective videos to address HIV stigma.

These findings imply that media, even short and one-time viewings, may improve HIV-related stigma among individuals in key populations in the short-run. Although this change does not represent a fundamental societal or institutional shift in attitudes or beliefs, this window of change may be adequate for increasing the acceptance of HIV services such as counseling and testing in the hours and days after viewing. Future studies might assess the impact of delivering low-cost and simple media in clinic waiting rooms, particularly targeting most at-risk communities with the aim of increasing the number of people who accept HIV testing and counseling.

## Conflict of Interest Statement

The authors declare that the research was conducted in the absence of any commercial or financial relationships that could be construed as a potential conflict of interest.
